# Neuromodulation of Chemical Synaptic Transmission Driven by THz Photons

**DOI:** 10.34133/research.0010

**Published:** 2022-12-19

**Authors:** Xiaoxuan Tan, Yuan Zhong, Ruijie Li, Chao Chang

**Affiliations:** ^1^ Innovation Laboratory of Terahertz Biophysics, National Innovation Institute of Defense Technology, Beijing 100071, China.; ^2^ Astronaut Center of China, Beijing 100084, China.; ^3^ Department of Engineering Physics, Tsinghua University, Beijing 100084, China.; ^4^ Brain Research Center and State Key Laboratory of Trauma, Burns, and Combined Injury, Third Military Medical University, Chongqing 400038, China.

## Abstract

Postsynaptic currents of chemical synapse are modulated by multitudinous neurotransmitters, such as acetylcholine, dopamine, glutamate, and γ-aminobutyric acid, many of which have been used in the treatment of neurological diseases. Here, based on molecular dynamics simulations and quantum chemical calculation, we propose that 30- to 45-THz photons can resonate with a variety of typical neurotransmitter molecules and make them absorb photon energy to activate the transition to high energy state, which is expected to be a new method of neural regulation. Furthermore, we verified the calculated results through experiments that THz irradiation could substantively change neuronal signal emission and enhance the frequency, amplitude, and dynamic properties of excitatory postsynaptic current and inhibitory postsynaptic current. In addition, we demonstrated the potential of neural information regulation by THz photons through 2-photon imaging in vivo. These findings are expected to improve the understanding of the physical mechanism of biological phenomena and facilitate the application of terahertz technology in neural regulation and the development of new functional materials.

## Introduction

Since the 18th century, paradigms used to describe the nature of light and its interaction with matter have gone through Newton’s theory of light particles, Maxwell’s wave paradigm of light, and Planck and Einstein’s theory of photons, until de Broglie unified matter and light and proposed the wave-particle duality of light. When there is no strong interaction between photons and matter, photons behave like a wave, and when it has a strong interaction with matter (absorption or emission), photons act more like particles [1–3].

Along come more and more scientists who believed that consciousness was a matter [4]. The generation, transmission, and coupling of neural signals should conform to electromagnetic fields and quantum theory. The quantum energy states of ions and molecules in the nervous system are important factors for signal generation, amplification, and coupling. The storage of biological information should be based on molecular quantum states [5,6]. There are also those who believe that consciousness has nothing to do with quantum mechanics. They argue that the brain is a wet, warm system in which decoherence destroys the quantum superpositions of neuron firing much faster than we thought, preventing our brains from acting as quantum computers [7]. However, recent studies have shown that terahertz wave irradiation in the low absorption band of water can significantly change the waveform and emission frequency of neuronal action potential (AP) [8], and effectively improve the learning ability of animals ~50%, while ultraviolet radiation has not observed similar effects [9]. This suggests that the regulation of the neural information by the terahertz wave is a thermal effect, but the specific THz photons may be dominant in the physiological process.

According to the traditional viewpoint of molecular physics, biophoton is generated during the transition of living matter from high energy state in the process of biological system metabolism. However, due to the extremely weak energy of biophoton radiation and the lack of sensitive detection methods, the relevant mechanism and significance have not been clarified [10–14]. The concept of a photon as a particle also leads many researchers to use it as a “massless reagent,” which can react with molecules and be absorbed after collision, leading to a reaction of *R* + *hv* → *R, and the number of photons can be calculated according to *E* = *N*_0_*hv* = *N*_0_(*c*/*λ*) [15]. In order to study the interaction between terahertz waves and biological systems, a large number of studies have been reported, confirming that the interaction between terahertz waves and various substances is universal, and it can affect the physical and chemical properties of both organic and inorganic materials. Terahertz is important for many biotechnology- and engineering-related phenomena, such as simulation studies on the influence of terahertz on water filtration [16], DNA unwinding [17,18], ion channel permeability [19], and myelin sheath information transduction [20–22]. For example, recent theoretical studies revealed that THz waves can promote the unwinding of DNA due to the resonance of THz waves with the vibration of DNA bases [17], and it can also induce structural transition and superpermeation of confined monolayer water [16]. Besides, there were also studies that proposed that myelin can serve as an infrared dielectric waveguide because the myelin sheath possesses a ≈2-fold higher refraction index compared to the outer medium or the inner axon with a certain mid-infrared to terahertz spectral range. Nevertheless, the quantum mechanisms underlying their molecular dynamics and chemical reactivity are not fully understood. At the same time, the unique optical, electronic, and biological properties of the different frequencies of terahertz also need to be translated into practice in experiments.

**Fig. 1.**
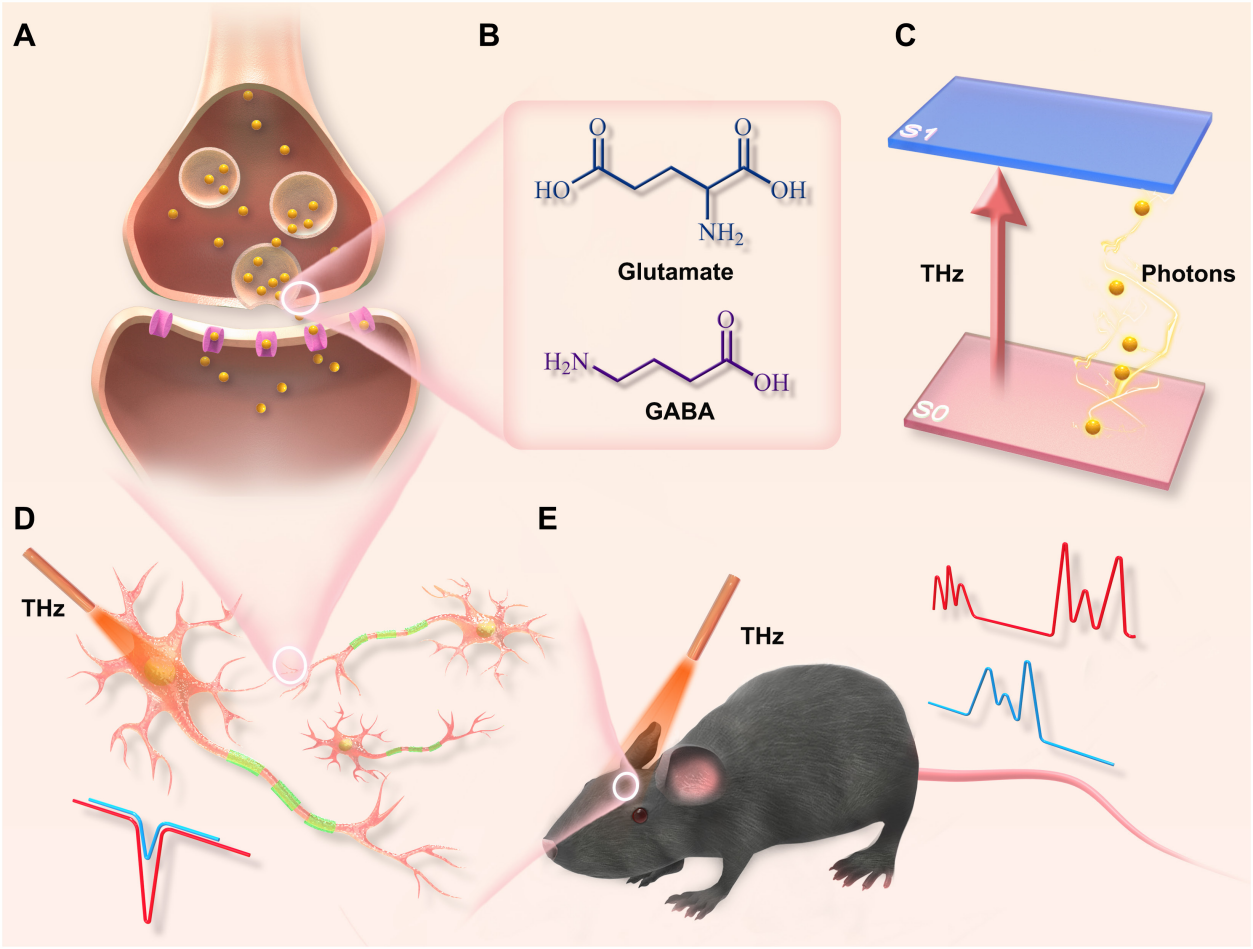
(A) Synaptic connection diagram. (B) Molecular structure of glutamate and GABA. (C) THz can stimulate the neurotransmitter to release photons. (D) THz photons enhance postsynaptic currents in vitro. (E) THz photons alter neuronal excitability in vivo (in the actual experiment, the brain of the mice was cut open). The blue and red traces in (D) and (E) represent synaptic and Ca^2+^ signals from recorded neurons.

As we know, in addition to the AP that has been shown to be mediated by THz photons, communication between neurons, known as synaptic transmission, also plays an important role in neural signal processing and transmission [23–28], and the intensity and precision of the quantum postsynaptic potential are very important to satisfy the conduction velocity of neuron signals [29]. Activation and inactivation of neurotransmitters directly affect postsynaptic currents of chemical synapse, so neurotransmitters provide an excellent option to explore the photonic properties of neural information. Here, we calculated the properties (infrared and Raman spectra, molecular orbitals, excitation energy, fluorescence emission energy, etc.) of 2 typical neurotransmitters [glutamate and γ-aminobutyric acid (GABA)] via density functional theory (DFT) and time-dependent density functional theory (TDDFT) and found the terahertz band with characteristic response. Based on this, we used the independently developed quantum cascade THz irradiation system, confirming that THz photons in the corresponding frequency band can significantly change the excitability of neurons and improve the transmission efficiency of nerve signals through the patch-clamp and 2-photon imaging in vivo technology. As shown in Fig. 1A to E, these results provide conclusive evidence that photons are indeed one of the key forms of biological information transmission and indicate that terahertz wave modulation (THM) has great application potential in the field of biological information regulation.

## Results and Discussion

### The ground state spectra of neurotransmitters

Infrared spectra and Raman spectra are often combined to obtain molecular vibration information. When polar groups and asymmetric molecules absorb infrared radiation during the vibration process, the dipole moments change, and the molecule transitions from the ground state to the excited state, resulting in the infrared absorption spectrum. In addition, the external electric field of the incident photon will cause the deformation of the electron shell of the molecule, and the relative movement of the positive and negative charge centers of the molecule will form the induced dipole moment, resulting in polarization phenomenon, which is the formation principle of Raman spectrum [30–33]. In density functional (DFT) calculation, we use a harmonic oscillator model to calculate the vibration frequency and calculate the second derivative of energy with respect to the coordinate to get the corresponding resonance potential function.

**Fig. 2.**
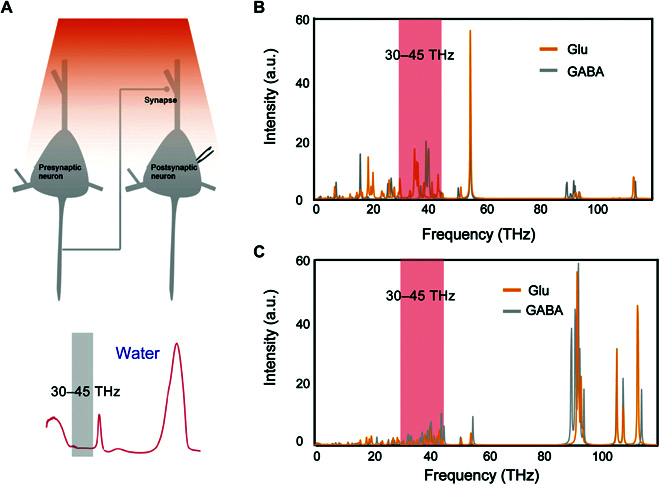
Mechanism by which terahertz enables neurotransmitters to transition to an excited state. (A) Diagram of synaptic connections. (B) Infrared spectra of 2 neurotransmitters and water. (C) Raman spectra of 2 neurotransmitters. The orange curves represent the excitatory neurotransmitter glutamate, and the gray curves represent the inhibitory neurotransmitter GABA. a.u., arbitrary units.

The various functional neural networks formed by synaptic connections between neurons are mainly maintained by a balance between glutamate-mediated excitation and GABA-mediated inhibition [21–24]. Therefore, we calculated the spectra of glutamate and GABA. Previous studies [8] have shown that water has strong absorption in parts of the terahertz band, meaning that careful wavelength selection is critical for neurotransmitter regulation (Fig. 2A). As shown in Fig. 2B, both neurotransmitters have strong resonance absorption peaks at 30 to 45 THz, while water has very weak absorption of THz wave at this frequency band, so 30 to 45 THz can act directly on the target molecule without being absorbed by the abundant water in the biological system. In addition, according to Raman spectra (Fig. 2C), the polarization changes of the 2 neurotransmitters caused by 30 to 45 THz are very small, indicating that the physical principle of activation by THz photons can be attributed to the characteristic vibration between electromagnetic wave and neurotransmitter, and the neurotransmitter absorbs radiation and produces electronic state transition.

### Neurotransmitter excitation process

**Fig. 3.**
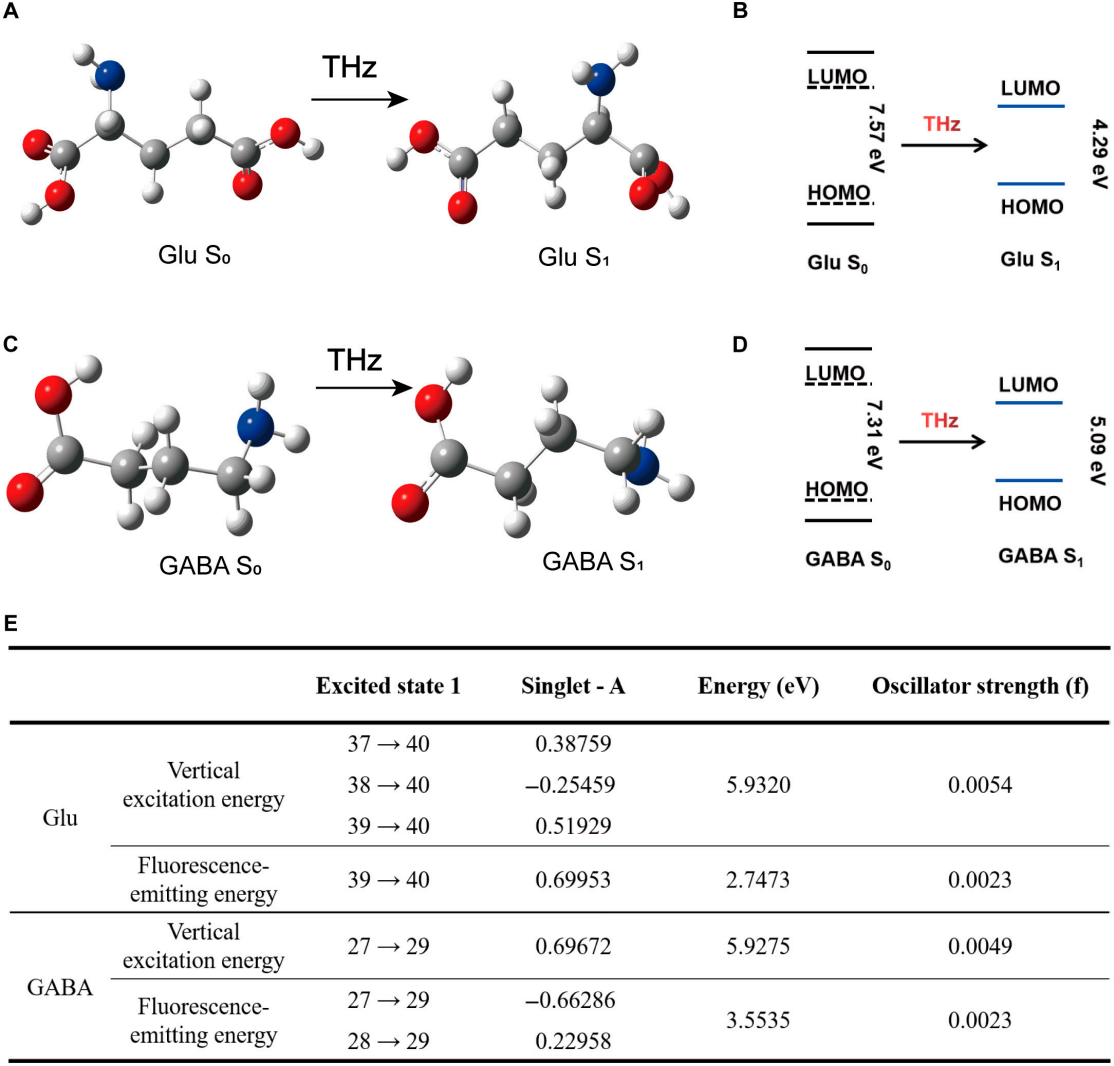
THz activates neurotransmitters and the mechanism illustrations. (A) DFT and TDDFT optimized structures for the ground and excited states of glutamate and (B) corresponding molecular orbital energies. (C) DFT and TDDFT optimized structures for the ground and excited states of GABA and (D) corresponding molecular orbital energies. (E) Vertical excitation energy and fluorescence emission energy of glutamate and GABA.

Neurotransmitters respond well to terahertz waves at specific frequencies, including strong absorption of 30 to 45 THz without water interference and subsequent molecular relaxation, encouraging further investigation of the mechanisms involved. First, by using DFT and TDDFT calculations, we calculate the geometric structures in the ground state (S_0_) and the first excited state (S_1_) to decipher the different conformations of the molecule under study. (Fig. 3A and C and Figs. S7 and S8).

The molecular orbitals involved in the S0–S1 transitions were also calculated. In orbitals of glutamate, the highest occupied molecular orbital (HOMO) and the lowest unoccupied molecular orbital (LUMO) were all spread on the -NH_3_ group. In orbitals of GABA, the HOMO was spread on the -NH_3_ group, while the LUMO was primarily spread on the -COOH group. The HOMO energy increased, while the LUMO energy decreased with the strong absorption of THz photons, resulting in a significant decrease in the HOMO–LUMO gap. As a result, the HOMO–LUMO gap of glutamate decreased from 7.57 eV to 4.29 eV, and the HOMO–LUMO gap of GABA decreased from 7.31 eV to 5.09 eV (Fig. 3B and D). Thus, the small HOMO–LUMO energy gap promotes subsequent molecular relaxation and photon liberation. Meanwhile, vertical excitation energy and fluorescence-emitting energy of 2 neurotransmitters were calculated (Fig. 3E).

Excitingly, the excited states S1 of glutamate and GABA also have strong absorption (Figs. S1 to S4) at 30 to 45 THz, so THz photons are promising for simultaneous neurotransmitter regulation and rapid detection at the femtosecond scale by pulse-probe flash spectroscopy [34].

### THz photons enhances postsynaptic currents in vitro

We used an independently designed and manufactured pulsed quantum cascade laser as the THz light source in this study (see the Supplementary Materials for details). A THz fiber (core diameter, 240 μm; numerical aperture, 0.27) delivered the THz photons to the target region of the brain slice. We delivered the THz with the following parameters: average irradiation power, ~30 mW; pulse width, 2 μs; repetition rate, 200 kHz.

**Fig. 4.**
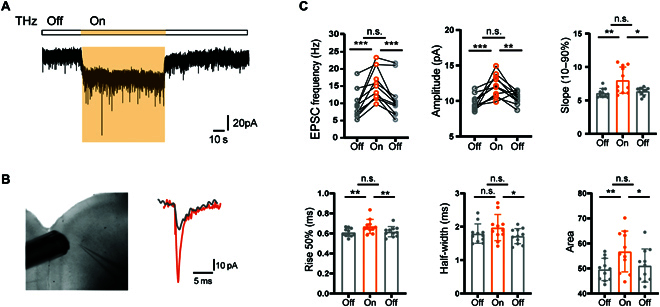
THz enhanced the EPSC. (A) Typical response during terahertz irradiation (shaded in orange). (B) Left: Physical image of THz and whole-cell recording from a PN in prefrontal cortex (PFC) slice. Right: Example EPSC responses to THz. Gray, control (Ctrl); orange, THz. (C) Group data showing changes in frequency, amplitude, slope, rise 50% time, half-width, and area upon THz. *P < 0.05, **P < 0.01, and ***P < 0.001. Paired Student’s *t* test and repeated-measures one-way ANOVA. Error bars represent SEM. n.s., not significant.

To examine the effect of THz on postsynaptic currents, we performed whole-cell voltage patch-clamp recording from layer 5 pyramidal neurons (PNs) in acute slices of mouse prefrontal cortex (Fig. 4B and Fig. S16). Close examination of the excitatory postsynaptic current (EPSC) revealed a dramatic change in frequency and amplitude (Fig. 4A and B) and the change was readily reversible (Fig. 4C). The EPSC frequency substantially increased by ~64%, from 9.53 ± 1.19 to 15.65 ± 1.39 Hz (*n* = 11 cells; *t*_10_ = 4.956; *P* = 0.6 × 10^−3^, paired Student’s *t* test), and the peak amplitude increased by ~46%, from 9.75 ± 0.31 to 12.12 ± 1.55 pA (*n* = 12 cells; *t*_11_ = 4.681; *P* = 0.7 × 10^−3^, paired Student’s *t* test). We also counted the changes of EPSC frequency in different irradiation periods and found that the effect of THz on EPSC frequency gradually weakened with time (Fig. S15). In addition, THM significantly changed the kinetic properties of EPSC, and the area (from 49.64 ± 4.45 to 56.79 ± 8.15 pA·ms; *n* = 11 cells; *P* = 0.72 × 10^−2^, paired Student’s *t* test) and slope (from 6.11 ± 0.67 to 8.03 ± 1.96 pA·ms; *n* = 11 cells; *t*_10_ = 3.602; *P* = 0.48 × 10^−2^, paired Student’s *t* test) were increased during THM. However, the rise time (from 2.52 ± 0.11 to 2.46 ± 0.16 ms; *n* = 12 cells; *t*_11_ = 1.857; *P* = 0.09, paired Student’s *t* test), decay time (from 4.99 ± 0.72 to 4.47 ± 0.73 ms; *n* = 12 cells; *t*_11_ = 2.985; *P* = 0.012, paired Student’s *t* test), and half-width (from 1.79 ± 0.29 to 1.97 ± 0.39 ms; *n* = 11 cells; *t*_10_ = 1.761; *P* = 0.11, paired Student’s *t* test) showed no significant change during THM, although the time to rise by 50% increased (from 0.61 ± 0.04 to 0.67 ± 0.07 ms; *n* = 11 cells; *t*_10_ = 3.320; *P* = 0.77 × 10^−2^, paired Student’s *t* test; Fig. 4C and Fig. S9).

Then, we conducted a new set of experiments and tested the effect of the same power (10 mW) of THz and Blu-ray on the EPSC separately. As shown in Fig. S11, Blu-ray irradiation also enhanced the frequency of EPSC (~2.08 times; *n* = 8 cells; *t*_7_ = 4.697; *P* = 0.0022, paired Student’s *t* test), but the effect was far less obvious than THz (~5.51 times; *n* = 6 cells; *t*_5_ = 4.390; *P* = 0.0071, paired Student’s *t* test). Besides, THz significantly changed the amplitude, slope, and area of EPSC, but these parameters did not change significantly under blue light irradiation, indicating that there was a nonthermal effect of EPSC in response to THz and the effect produced by THz photons is specific, although it could not rule out a thermal effect. We also tested the effect of THz on evoked currents, which was consistent with the amplitude change of spontaneous postsynaptic currents, increasing from 77 ± 4.09 to 147 ± 12.05 pA (*n* = 6 cells; *t*_5_ = 6.452; *P* =0.0013, paired Student’s *t* test; Fig. S12).

**Fig. 5.**
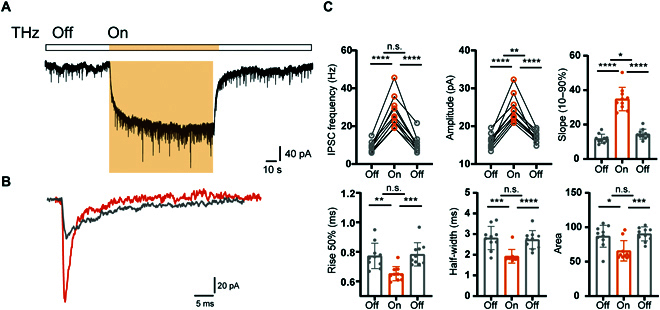
THz enhanced the IPSC. (A) Typical response during terahertz irradiation (shaded in orange). (B) Example IPSC responses to THz. Gray, control (Ctrl); orange, THz. (C) Group data showing changes in frequency, amplitude, slope, rise 50% time, half-width, and area upon THz. *P < 0.05, **P < 0.01, ***P < 0.001, and ****P < 0.0001. Paired Student’s *t* test and repeated-measures one-way ANOVA. Error bars represent SEM.

Next, we examined the effects of THz on inhibitory postsynaptic currents (IPSCs). In agreement with calculated results, THz photons cause many significant changes in IPSC (Fig. 5A and B). The frequency and peak amplitude increased by ~209% (from 8.87 ± 2.81 to 27.44 ± 8.27 Hz; *n* = 10 cells; *P* <10^−4^, paired Student’s *t* test) and ~54% (from 15.89 ± 1.89 to 24.47 ± 3.59 pA; *n* = 10 cells; *P* <10^−4^, paired Student’s *t* test), respectively; the 10% to 90% slope of the IPSC rising phase increased from 11.75 ± 2.61 to 34.83 ± 6.78 pA·ms (*n* = 10 cells; *P* <10^−4^, paired Student’s *t* test), and the area decreased from 86.92 ± 15.91 to 65.74 ± 14.84 pA·ms (*n* = 10 cells; *P* = 0.9 × 10^−3^, paired Student’s *t* test). Besides, other dynamic properties of IPSC such as rise 50% time (from 0.77 ± 0.09 to 0.65 ± 0.05 ms; *n* = 10 cells; *t*_9_ = 4.478; *P* = 0.15 × 10^−2^, paired Student’s *t* test) and half-width (from 2.81 ± 0.56 to 1.92 ± 0.33 ms; *n* = 10 cells; *t*_9_ = 6.002; *P* = 0.2 × 10^−3^, paired Student’s *t* test) also changed significantly during THz application (Fig. S10). Similar to EPSC, these changes of IPSC were also reversible and reproducible. However, some of the dynamic properties of IPSC show opposite changes to those of EPSC, such as rise 50% time, half-width, and area (Fig. 5C and Fig. S10). We speculate that this may be due to the different types and numbers of specific bonds that resonate with different neurotransmitters caused by 30- to 45-THz photons, and the change of vibration mode and frequency is also different, which is manifested at the cellular level by the different dynamic properties of postsynaptic currents. By recording miniature EPSC (mEPSC) and miniature IPSC (mIPSC), we also found that THz photons changed the presynaptic quantal release changes (Figs. S13 and S14). These experiments above together establish a basic proof of principle for THz photons in vitro, demonstrating that THz energy delivery indeed induces neurotransmitter activation in the absence of any exogenous materials.

### THz photons alters neuronal excitability in vivo

**Fig. 6.**
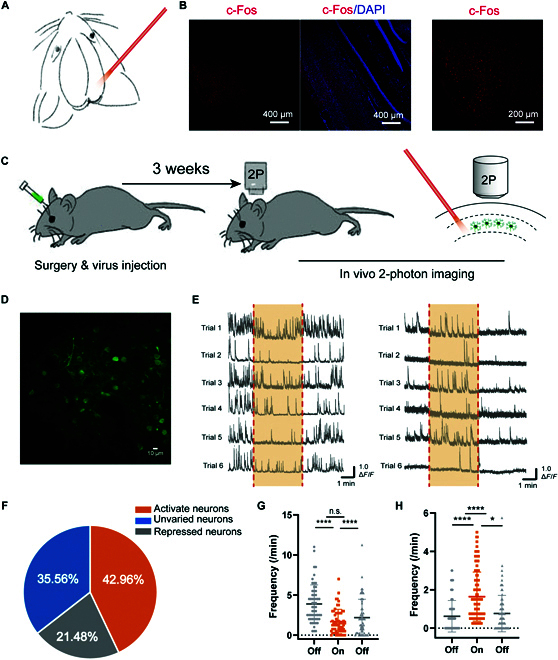
THz photons alter neuronal excitability in vivo. (A) General scheme for targeted THz photon delivery to mouse brain in vivo. (B) Left: Confocal image of a postmortem slice near the THz target spot. Right: Magnified view of the left image (c-Fos is a commonly used marker of neuronal activation). (C) Cartoon showing the general scheme for the in vivo 2-photon imaging experiments. (D) An example in vivo 2-photon image (averaged 100 frames). (E) Ca^2+^ activity traces of 12 example neurons. Excited neurons were suppressed (left) and neurons that were not excited were activated (right). (F) Proportion of neurons with 3 patterns of change, 270 neurons from 5 animals. (G) Changes in frequency of Ca^2+^ signaling in inhibited cells. (H) Changes in frequency of Ca^2+^ signaling in excited cells. *P < 0.05, **P < 0.01, ***P < 0.001, and ****P < 0.0001. Paired Student’s *t* test and repeated-measures one-way ANOVA. Error bars represent SEM.

Motivated by the above results, we tested whether THz photons affects neural signals in vivo. As described in a previous study [9], we first placed the fiber tip closely above the cortical surface following craniotomy, and the irradiation time was 60 s (Fig. 6A). After THz delivery, we performed immunohistochemistry for c-Fos, a widely used molecular marker of neuronal activation. As shown in Fig. 6B, a large number of neurons in the target area were activated, and the c-Fos–positive cells can reach layer 5 of the cortex.

We next employed 2-photon Ca^2+^ imaging to visualize live neuronal population activities during THz application (Fig. 6C). Sensory cortex neurons were labeled with a genetically encoded Ca^2+^ indicators, AAV-GCamp6f. After virus expression, we performed real-time (30 Hz) 2-photon imaging over the entire course of THz application (2 min before, 4-min THz irradiation, 2 min after; Fig. 6D). An example animal showed that THz application reliably changed Ca^2+^ transients during the application time window over different cells. Similar to previous findings that THz photons enhance both EPSC and IPSC, imaging results showed that the excitability of some cells increased and some decreased during THz application (Fig. 6E). Interestingly, within the same cell type, the pattern of change seemed to be consistent, with cells that were excited being suppressed and cells that were not excited being activated.

Out of a total of 270 neurons from 5 animals, we found 116 THz-activated neurons (from 0.62 ± 0.08 to 1.64 ± 0.12 per minute; *n* = 116 cells; *t*_115_ = 15.57; *P* <10^−4^, paired Student’s *t* test) and 58 THz-suppressed neurons (from 3.89 ± 2.37 to 1.69 ± 1.52 per minute; *n* = 58 cells; *t*_57_ = 12.11; *P* <10^−4^, paired Student’s *t* test; Fig. 6F). The activity level for the THz-activated neurons recovered after the THz window, returning to the same level as before irradiation (from 0.62 ± 0.08 to 0.76 ± 0.09 per minute; *n* = 116 cells; *t*_115_ = 1.963; *P* = 0.052, paired Student’s *t* test; Fig. 6G), and at the same time, although the excitation level of the inhibited cells recovered after irradiation (from 1.69 ± 1.52 to 2.18 ± 2.30 per minute; *n* = 58 cells; *t*_57_ = 2.280; *P* = 0.026, paired Student’s *t* test; Fig. 6H), it did not reach the pre-irradiation level (from 3.89 ± 2.37 to 2.18 ± 2.30 per minute; *n* = 58 cells; *t*_57_ = 7.245; *P* <10^−4^, paired Student’s *t* test; Fig. 6H). Note that the proportion of THz-changed neurons detected in these 2-photon Ca^2+^ imaging experiments was 64.44% (including 42.96% activated neurons and 21.48% suppressed neurons), which was lower than that in the patch-clamp results (EPSC or IPSC increased in all tested cells). We speculate that this difference could result from the differences in test subjects, i.e., the synaptic connections between a large number of neurons are cut in the brain slice experiment, and the neural circuits or interactions between neurons are far less complex than in vivo. Nevertheless, our results from patch-clamp, c-Fos imaging, and 2-photon Ca^2+^ imaging are highly consistent in demonstrating that THz photons can change the activity of a substantial number of neurons in the targeted cortical region.

It is important to point out that although THM is similar to the well-known and widely deployed photodynamic therapy, it can reliably induce photon-triggered molecular responses in a precisely targeted and spatially confined volume of biological tissue in vivo, and the 2 are fundamentally different. Biocompatibility of nanofluorophores is an important concern for their in vivo application, but THM does not require injection of exogenous materials, and its effect depends entirely on the quantity and frequency of photons rather than the properties of encapsulated molecules. It has good stability and excellent biocompatibility, which facilitate clinical translation [35–37].

## Conclusion

In this study, we found that THz photons can modulate postsynaptic currents. It increases the frequency, amplitude, and kinetic properties of EPSC and IPSC, which is probably because THz photons can resonate with a variety of typical neurotransmitter molecules and make them absorb photon energy to activate the transition to high energy state. In addition, THz also increases the amplitude of mEPSC, mIPSC, and evoked currents. Control experiments with Blu-ray stimulation revealed that THz photons provide nonthermal modulation of chemical synaptic transmission. We further demonstrate that THz photons could change neuronal excitability. Therefore, THz photons with a specific wavelength could be a new method to modulate neuronal excitability and synaptic transmission directly in vitro and in vivo.

On the one hand, our results illustrate that THz photons could be used as a promising form of brain function modulation and brain disease therapy. Most previous studies explore the method of modulating brain activities by optogenetics, which is currently one of the best methods to modulate synaptic transmission. However, the optogenetics technique needs to introduce exogenous genes in the brain, and it shows little potential for applications in healthy humans. Our energy stimulation technique in this study is fundamentally different from the optogenetics because it does not require any introduction of a light-sensitive opsin into the neuronal cells.

On the other hand, our results provide a new theoretical approach to neuroscience research. There are many phenomena that theories of traditional neuroscience cannot explain, such as why nerve signals travel so fast in myelinated nerves. A simple flow of charged ions cannot keep up with that speed, while photons travel extremely fast. One study has established a physical model of neural high-frequency signal generation and transmission by quantum optics and electrodynamics methods [21,22]. Our results in this study provide experimental evidence for supporting the mechanism of infrared and terahertz neurotransmission

The assumption that consciousness can be understood as a state of matter, quantum or photon, has led to some interesting interdisciplinary questions, from neuroscience to materials science [5,6], condensed matter physics [17], and quantum mechanics [18,19]. When the frequency of photon is equal to the characteristic frequency of biological system, the photon may nonthermally modulate molecular-related functions and biological processes by influencing the electronic states and energies of biological macromolecules [3,12]. At the same time, the rotational and vibrational energy levels of biological macromolecules are mostly located in the terahertz band [38–41]. In summary, our results confirm the possibility of using quantum theory to characterize biological processes and the efficacy of THz photons for neural regulation, providing a new pathway for intervention in neural signal generation and transmission. A deeper understanding of the mechanism of biological information may provide us with subversive ideas and methods for developing new functional materials and exploring the unknown nature.

## Data Availability

Data are available from the corresponding author upon request.

## Supplementary Materials

Supplementary Materials Supplementary Materials and Methods Figs. S1 to S16 Tables S1 to S6 References
