# Supplementary material for: Neuromodulation of Chemical Synaptic Transmission Driven by THz Photons
Source: Research (Wash D C). 2022 Dec 19;2022:0010. doi: 10.34133/research.0010 (PMC11404318; doi:10.34133/research.0010)
Supplement: Supplementary Materials — and Methods Figs. S1 to S16 Tables S1 to S6 References [file research.0010.f1.docx]

Supporting Information

**Neuromodulation of synaptic transmission driven by THz photons**

**Neuromodulation of chemical synaptic transmission driven by THz photons**

*Xiaoxuan Tan^1,2,*^, Yuan Zhong^1,3^, Ruijie Li^4^, Chao Chang^1,*^*

^1^Innovation Laboratory of Terahertz Biophysics, National Innovation Institute of Defense Technology, Beijing 100071, China.

^2^Astronaut Center of China, Beijing 100084, China.

^3^Department of Engineering Physics, Tsinghua University, Beijing 100084, China.

^4^Brain Research Center and State Key Laboratory of Trauma, Burns, and Combined Injury, Third Military Medical University, Chongqing 400038, China.

Keywords: neuromodulation, terahertz, biophotons

This file includes:

Materials and Methods

Supplementary Figures 1 to 16

Supplementary Tables 1 to 6

References

**1.Materials**

The reagents chemicals used in the work were bought from sigma or Aladdin. AAV-gcamp purchased from braincase.

C57BL/6J mouse (2 months old) were provided by the Experimental Animal Center of Tsinghua University and the Laboratory Animal Center of the Third Military Medical University. All experimental procedures were performed in accordance with the approval of the Animal Care and Use Committee of Tsinghua University and the Third Military Medical University Animal Care and Use Committee and in accordance with institutional animal welfare guidelines.

**2.Methods**

**THz light source.** The quantum cascade terahertz laser were made based on a slightly-diagonal bound-to-continuum design, as describe in our previous studies[1,2]. The beam from the quantum cascade laser is coupled into a fiber (wavelength range: 3–17 μm, code: PIR600/700-100-FC/PC-FT-SP30, article number: AP12001, and type: step index multimode) by a coupler, the diameter of the fiber is 240 ± 10 μm and the numerical aperture (NA) was 0.35 ± 0.05.

**Mouse brain slice preparation.** Coronal slices of prefrontal cortex were obtained from C57/B6 mice (postnatal ~2 months). We anesthetized the mice with sodium pentobarbital (10 mg/kg of body weight, intraperitoneal injection) and sacrificed with decapitation. Brain tissues were then immediately dissected out and immersed in ice-cold oxygenated (95% O_2_ and 5% CO_2_) slicing solution in which 126 mM NaCl was substituted by 213 mM sucrose. Slices (250 μm in thickness) were cut with a microtome (VT-1200S, Leica, Germany) and incubated in the aerated normal ACSF (Table S5) and maintained at 34.5 ^o^C for 1.5 h. After incubation, slices were kept in the same solution at room temperature until use.

**Electrophysiological recordings from mouse neocortical slices.** Before recordings, the slices were moved to a recording room and perfused with a regular ACSF (0.9 ml/min). Cortical neurons were observed using an upright infrared differential interference contrast (IR-DIC) microscope (BX51WI, Olympus) equipped with a water-immersed objective (40x, NA 0.8). The patch pipette for somatic recordings had an impedance of 4-6 MΩ. The electrical signals were acquired using a Multiclamp 700B amplifier (Molecular Devices), digitized and sampled by Micro 1401 mk II (Cambridge Electronic Design) at 25 kHz using spike2 software.

**Synaptic transmission.** Neurons were held at -70 mV using the voltage clamp for recording EPSC and mEPSC, the patch pipettes were filled with normal internal solution Table S6 shown. Neurons were held at -60 mV using the voltage clamp for recording IPSC and mIPSC, the patch pipettes were filled with a Cs^+^-based internal solution containing (in mM): 2 MgCl_2_, 2 Na_2_ATP, 140 CsCl, 10 HEPES, and 10 EGTA (287 mOsm, pH 7.2 with CsOH). In addition, we included 1 mM Kyn in the bath solution to block excitatory currents to record IPSC, included 1µM TTX and 20 µM BMI in the bath solution to record mEPSC, included 1µM TTX , 10µM CNQX and 50µM AP-5 in the bath solution to record mIPSC.

**Immunohistochemistry.** In the c-Fos experiments, the animals were first injected with isoflurane (1.5%), and their heads were fixed after general anesthesia. Skull fenestration was performed on the sensory cortex. After the THz application, animals were kept under anesthesia for 90 minutes for c-Fos expression. Then 4% paraformaldehyde (PFA) was infused through the heart, and the brain was fixed in 4% PFA including 15% sucrose and refrigerated overnight at 4℃. The coronal sections (thickness: 44 µm) were prepared by a vibratome. The primary antibody is Anti-c-Fos (ABE457, Millipore) and the secondary antibody is Alexa Fluor 594 goat anti-rabbit 1:500 (A1 1012, Molecular Probes). DAPI(4’,6-diamidino-2-phenylindole, D9564, Sigma-Aldrich) stained the nuclei (1:10000 dilution). The sections were fixed to the slides with cover slides and imaged with scanning confocal microscope (TCS SP5, Leica).

**Virus injection.** Prior to surgery, the mice were immobilized in a stereotactic frame (RWD, Shenzhen, China) and under a-combination of xylazine (10mg/kg) anesthesia and ketamine (100mg/kg) analgesia. A calibrated glass microelectrodes connected to an infusion pump (micro 4, WPL, USA) was used to inject ~150 nl virus was injected using at a rate of 30 nl/min. The coordinates were defined as from the brain surface to dorsum-ventral (DV), from bregma to anterior-posterior (AP), and midline to medio-lateral (ML, in mm).

About 150 nl volume of AAV - Camkii - GCaMp6 - containing solution (~2×10^12^  infectious units/ml) was injected into sensory cortex slowly (AP: ~-1.5 mm, ML: ~2.0mm, DV: ~0.35mm) for imaging calcium activity.

**Two-photon Ca2+ imaging.** Two-photon calcium imaging was performed using a two-photon microscope (Nikon) equipped a mode-locked Ti: Sa laser (model “Mai-Tai Deep See”, Spectra Physics). Calcium activities were imaged using the 910 nm as excitation wavelength and the imaging was set at 30-Hz , the field-of-view (FOV) was set 200 µm × 200 µm with 512 × 512 pixels at frame rate.

**Quantum-chemical calculations.** Quantum-chemical calculations were performed to obtain the optimized geometrical parameters, vibrational frequencies and orbital energies of glu and GABA. The molecular geometries were investigated using density functional theroy (DFT) and time-dependent density functional theory (TDDFT) calculations. The PBE0 exchange correlation functional and 6-311G* basis set were employed in the DFT and TDDFT calculations. The polarized continuum model (PCM) was used for modeling solvation effects together with water as the solvent. The geometry optimization calculations were performed first to obtain the stable conformations of molecules and the lowest energy conformation was used for calculating results. The frequency of absorbing/emissive photon was identified by the vibrational mode where the atoms move towards the reaction direction. All calculations were performed by the Gaussian 16 software package and Gaussview 6.0 visualization program^[3-5]^.

**Data analysis.** We used Spike 2 and MATLAB (MathWorks, Bethesda, MD) to perform the data analysis. Summary data were presented as mean ± s.e.m and error bars in figures also represent s.e.m. Unless otherwise specified, groups were analyzed by two-tailed paired Student’s t-test if the data were paired. For more than two independent observations, RM-one-way ANOVA was applied. Differences were considered to be significant if P < 0.05. In vivo experiments, cell classification was based on calcium signal frequency, and neuronal excitability was considered to change when the frequency change exceeded 20%.

**
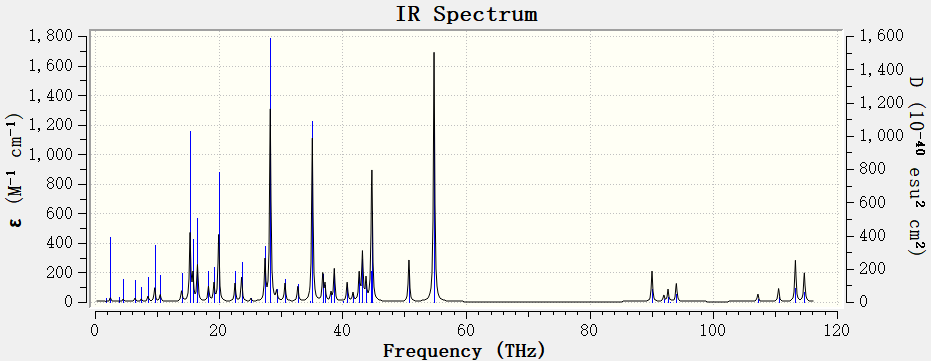
**

**Figure S1.** **Infrared spectrum of** **glutamate acid excited state.** Blue vertical line: positive correlation with infrared intensity; Black curve: absorption curve derived from Lorentz expansion based on infrared intensity and vibration frequency, full width at half maximum (FWHM = 8 cm^-1^)


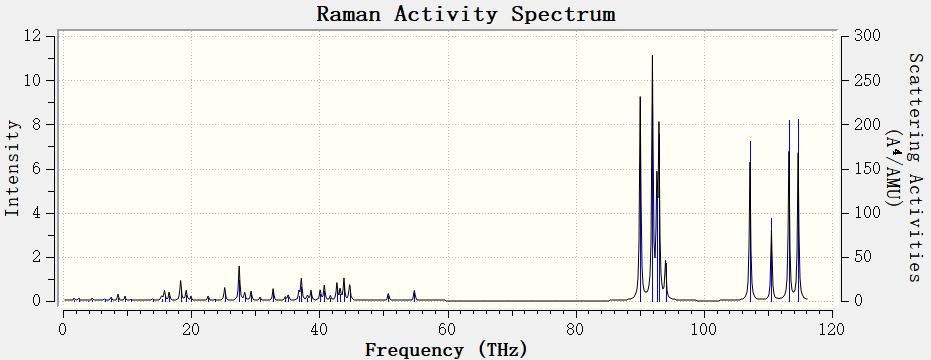


**Figure S2.** **Raman spectrum of glutamate excited state.** Blue vertical line: positive correlation with raman activity; Black curve: absorption curve derived from Lorentz expansion based on raman activity and vibration frequency, full width at half maximum (FWHM = 8 cm^-1^)


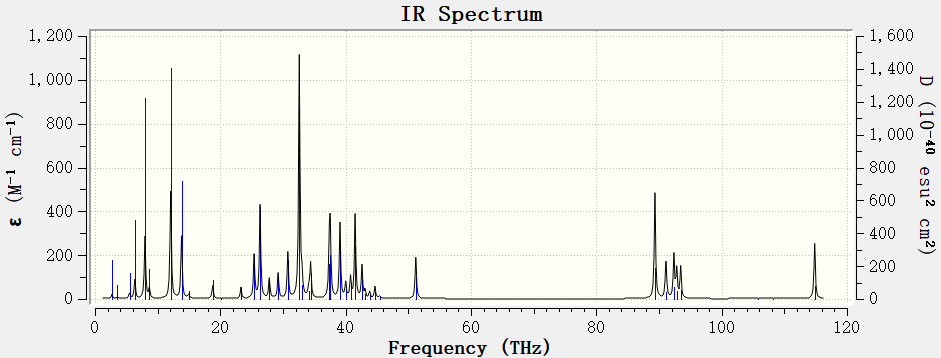


**Figure S3.** **Infrared spectrum of GABA excited state.** Blue vertical line: positive correlation with infrared intensity; Black curve: absorption curve derived from Lorentz expansion based on infrared intensity and vibration frequency, full width at half maximum (FWHM = 8 cm^-1^)


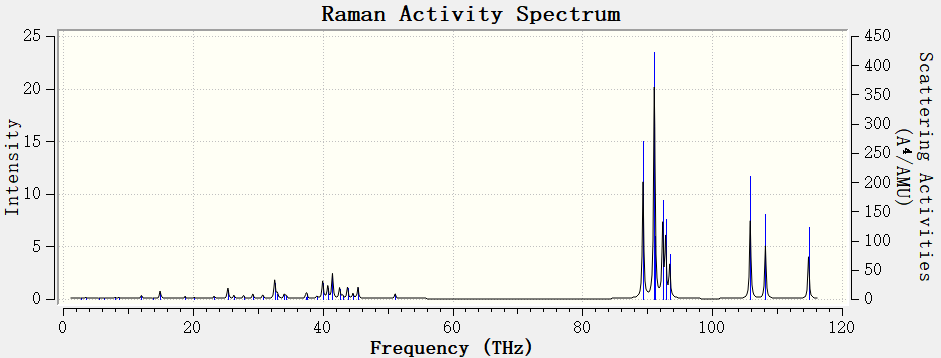


**Figure S4.** **Raman spectrum of GABA excited state.** Blue vertical line: positive correlation with raman activity; Black curve: absorption curve derived from Lorentz expansion based on raman activity and vibration frequency, full width at half maximum (FWHM = 8 cm^-1^)


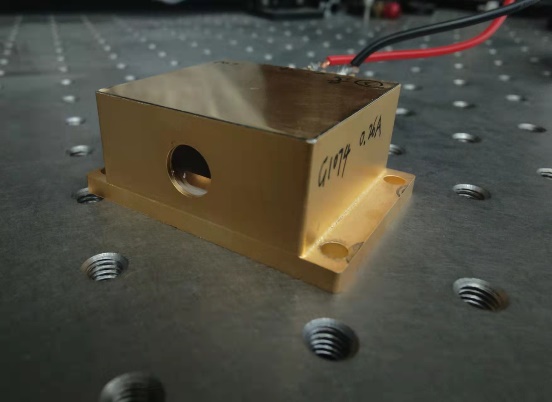

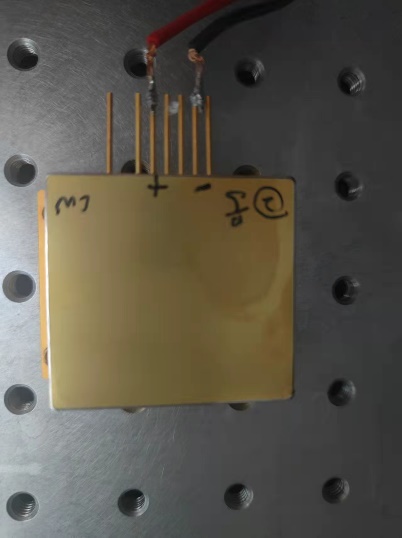


**Figure S5.** **Physical picture of quantum cascade laser.** The round hole on the left is the light outlet. Pins on the right from bottom to top are 1: temperature control negative pole; 2: quantum cascade laser negative pole; 3:10 kΩ; 4: 10 kΩ; 5: quantum cascade laser positive pole; 6: non-occupation; 7: do not install; 8: temperature control positive pole.


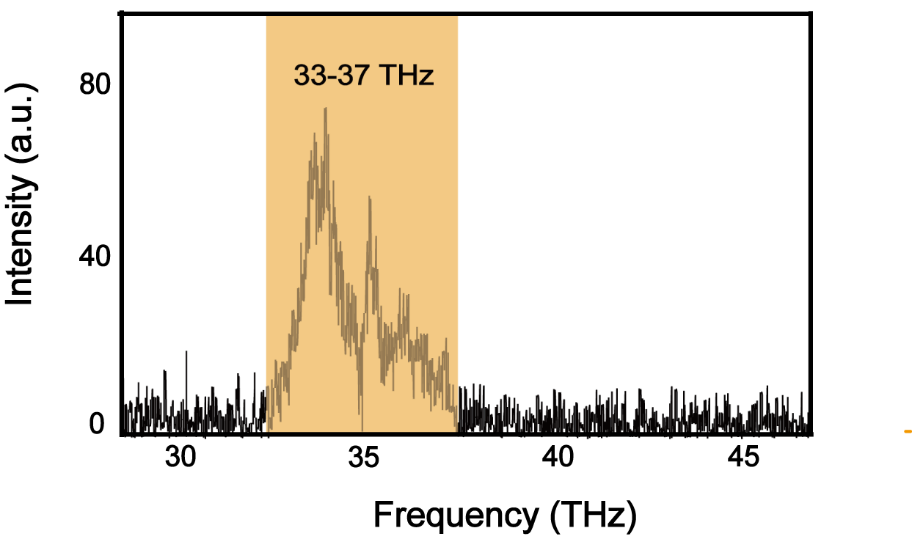


**Figure S6**. **Frequency band widths of laser**


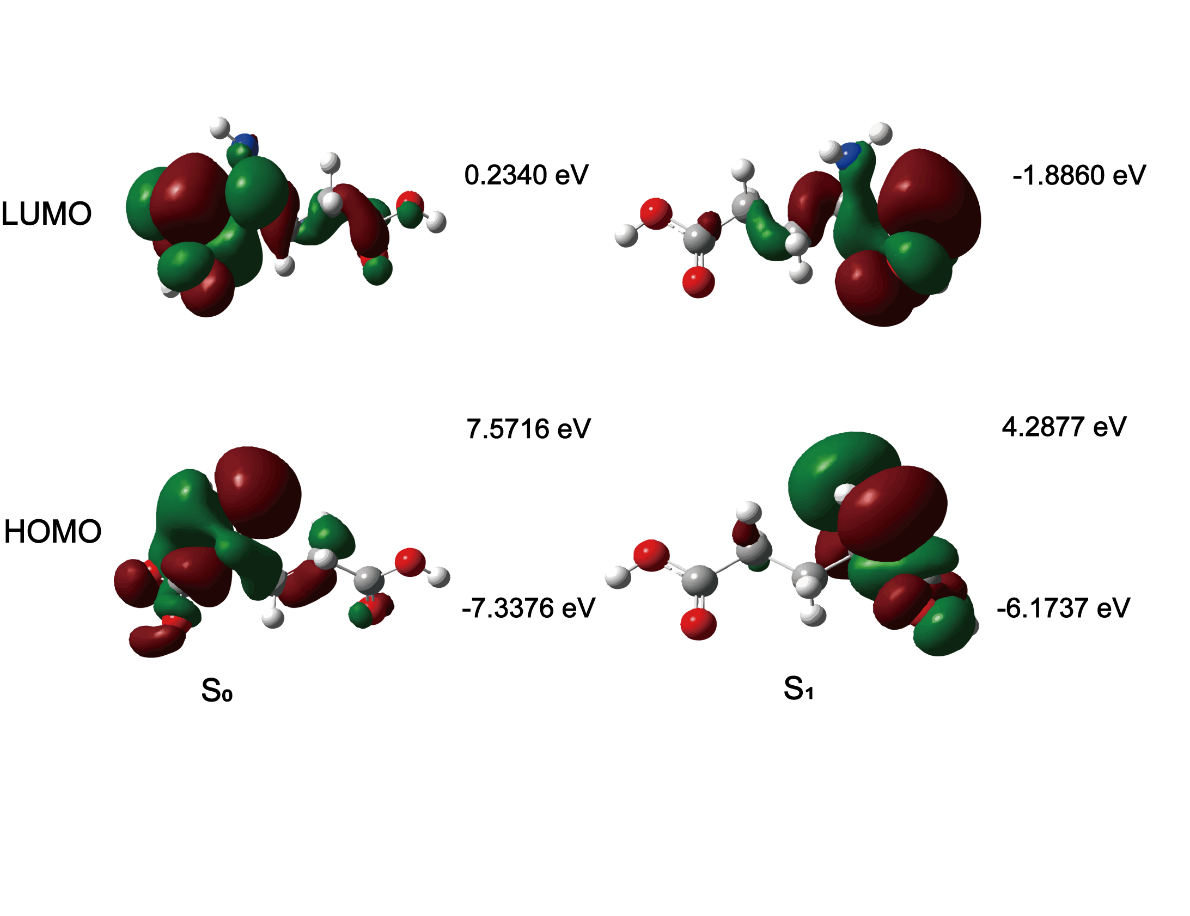


**Figure S7.** **The frontier molecular orbitals (MOs) of the ground-state S0 and excited state S1 for the glutamate.**


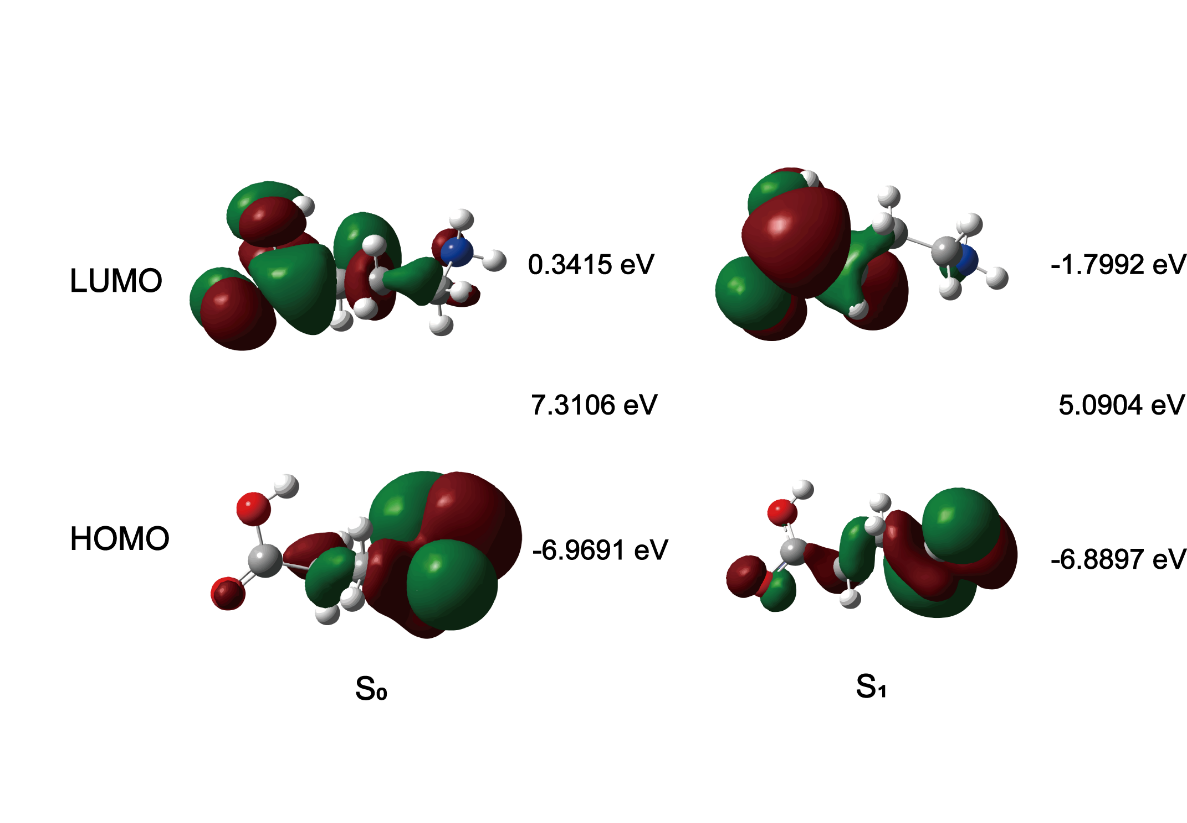


**Figure S8.** **The frontier molecular orbitals (MOs) of the ground-state S0 and excited state S1 for the GABA.**


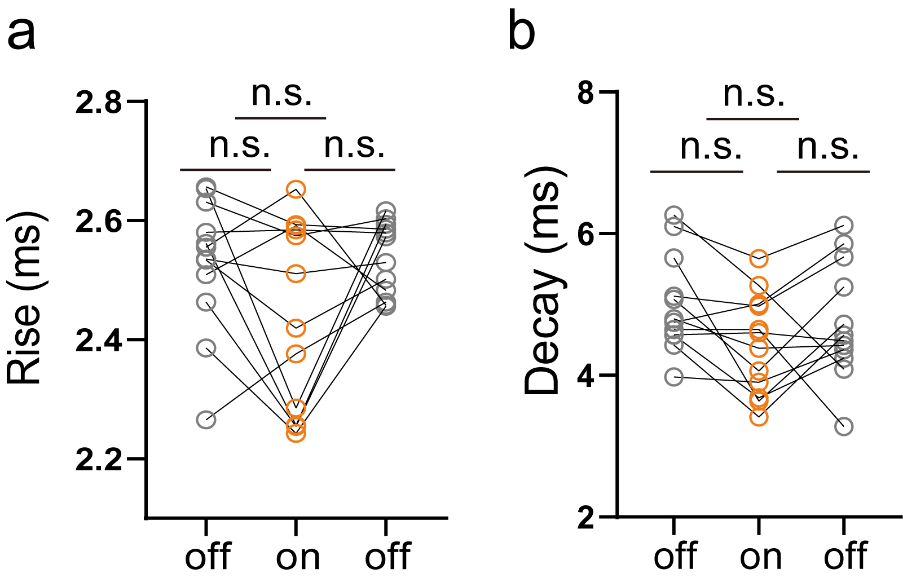


**Figure S9.** **Rise and decay time of EPSC**


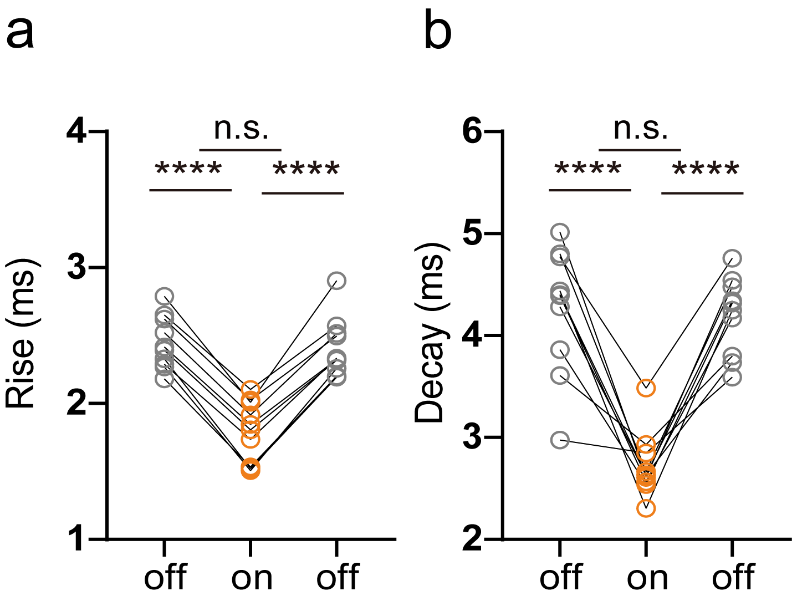


**Figure S10.** **Rise and decay time of IPSC**


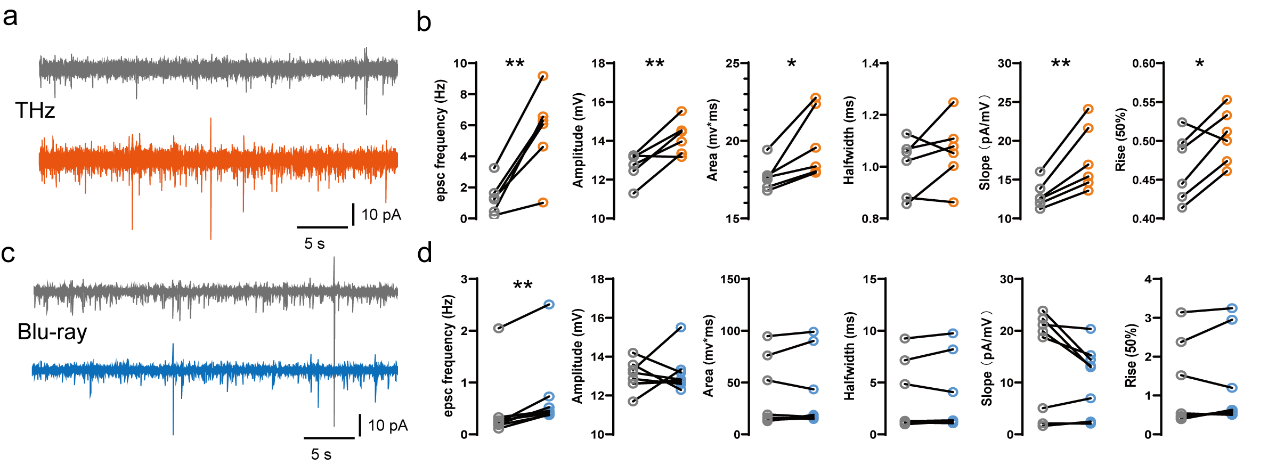


**Figure S11. THz and Blu-ray control experiments. a)** One consecutive trial of EPSC recording before (gray) and under THz irradiation (orange). **b)** Group data showing changes in frequency, amplitude, slope, rise 50% time, halfwidth and area upon THz, n=6. **c)** One consecutive trial of EPSC recording before (gray) and under Blu-ray irradiation (blue). **d)** Group data showing changes in frequency, amplitude, slope, rise 50% time, halfwidth and area upon Blu-ray, n=7. * and ** represent P < 0.05 and 0.01, respectively. Paired Student’s t-test. Error bars represent s.e.m.


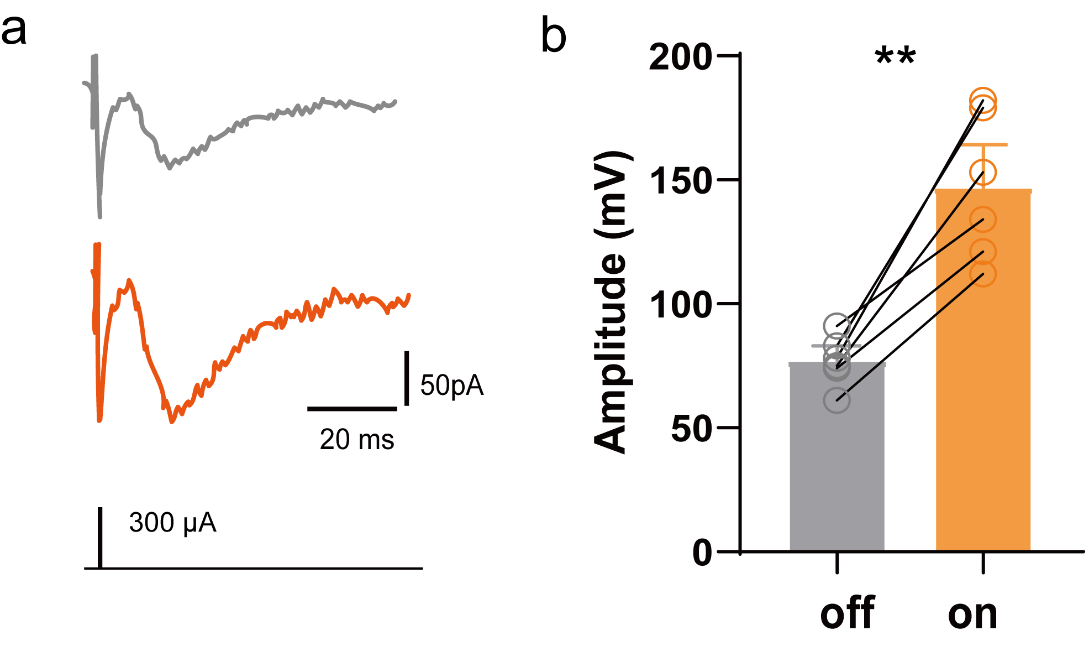


**Figure S12. a)** example evoked currents response to THz. Gray, control (off); orange, THz (on). **b)** data showing changes in amplitude of evoked currents upon THz. ** represent P < 0.01. Paired Student’s t-test. Error bars represent s.e.m.


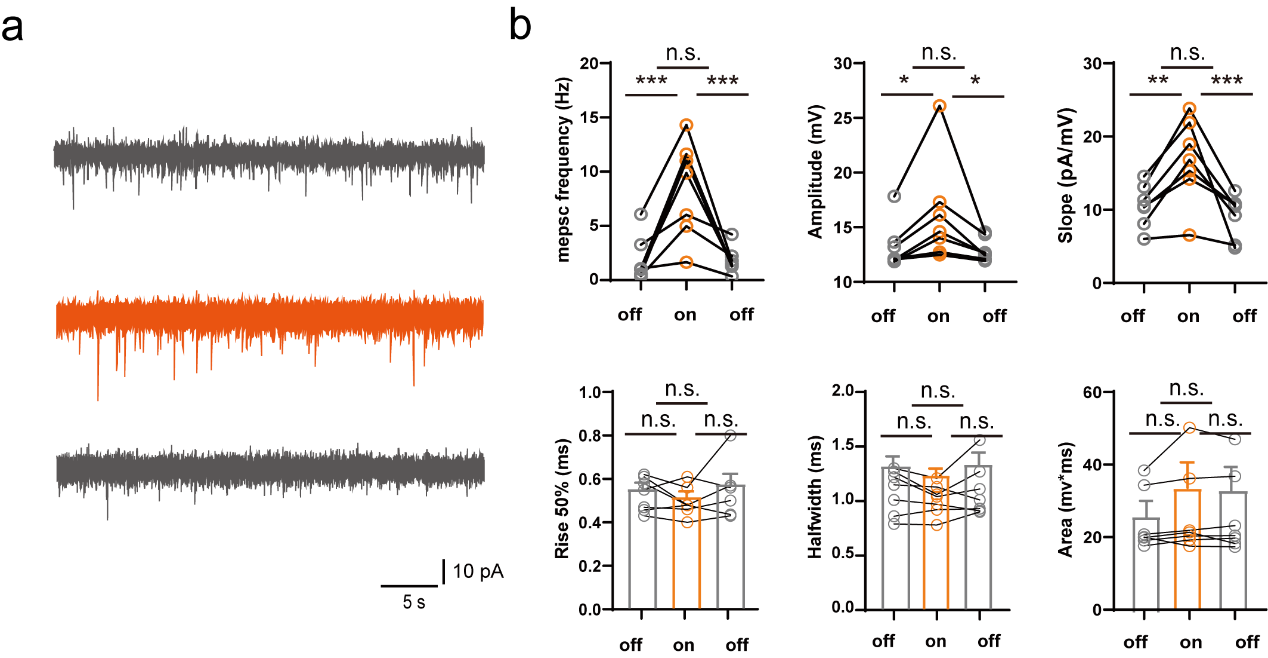


**Figure R13. THz enhanced the mini excitatory postsynaptic current (mEPSC). a)** Typical response before, during and after terahertz irradiation (from top to bottom). **b)** Group data showing changes in frequency, amplitude, slope, rise 50% time, halfwidth and area upon THz. *, ** and *** represent P < 0.05, 0.01 and 0.001, respectively. Paired Student’s t-test. Error bars represent s.e.m.


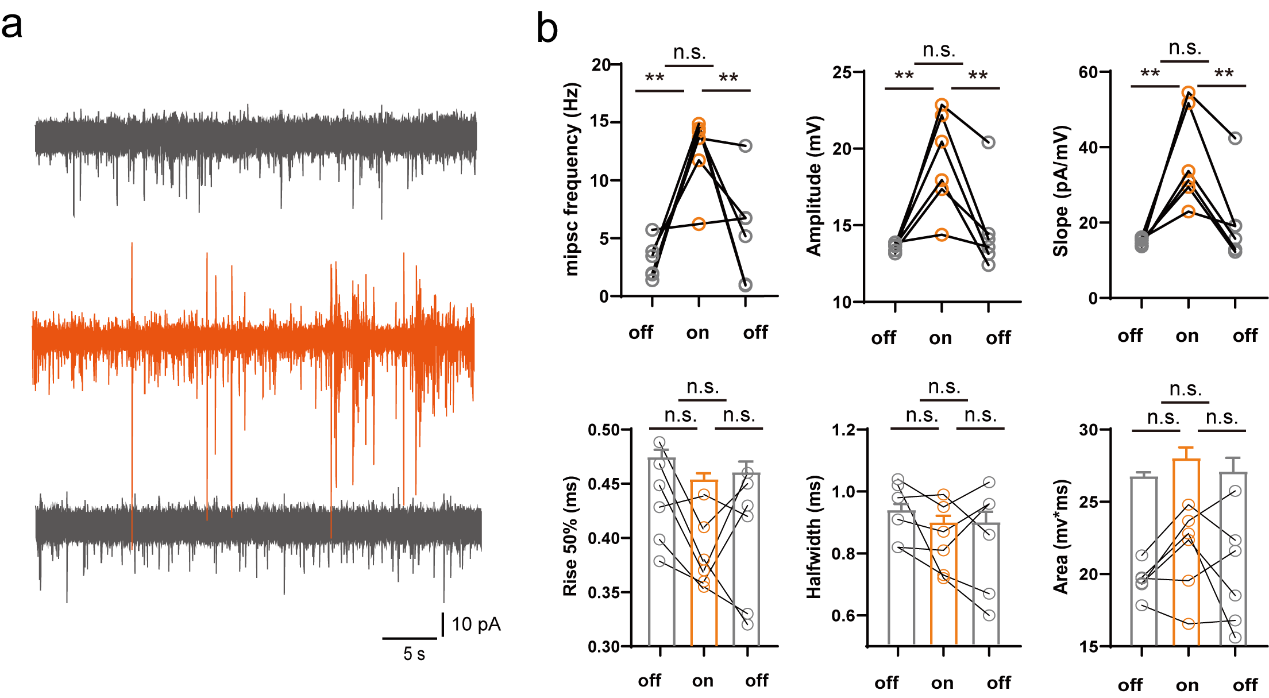


**Figure S14. THz enhanced the mini inhibitory postsynaptic current (mIPSC). a)** Typical response before, during and after terahertz irradiation (from top to bottom). **b)** Group data showing changes in frequency, amplitude, slope, rise 50% time, halfwidth and area upon THz. *, ** and *** represent P < 0.05, 0.01 and 0.001, respectively. Paired Student’s t-test. Error bars represent s.e.m.


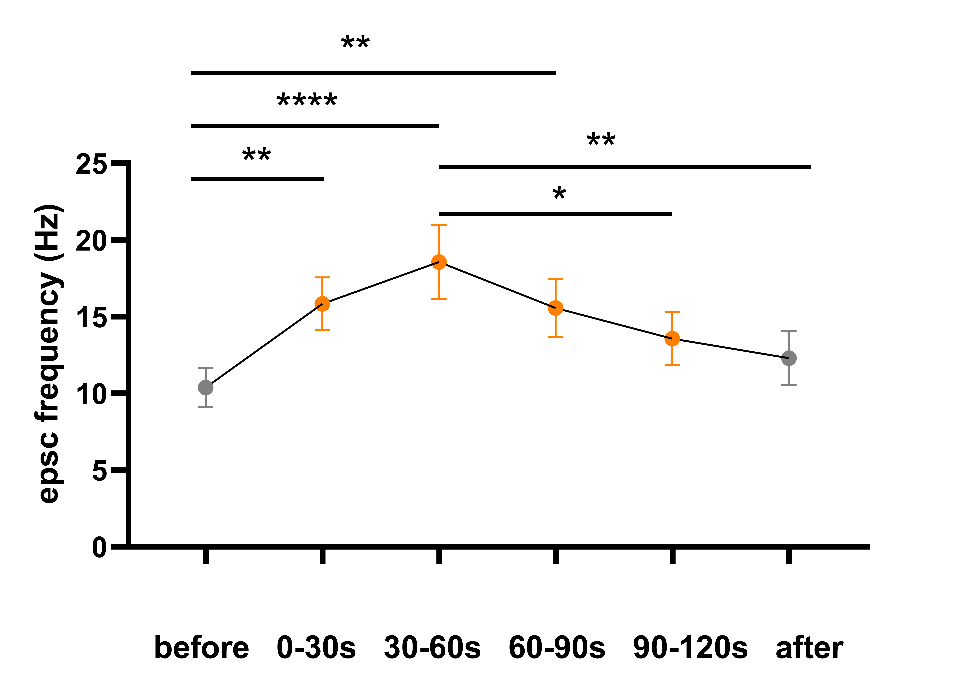


**Figure S15. The changes of epsc frequency in different irradiation periods.** *, ** and **** represent P < 0.05, 0.01 and 0.0001, respectively. RM-one-way ANOVA. Error bars represent s.e.m.


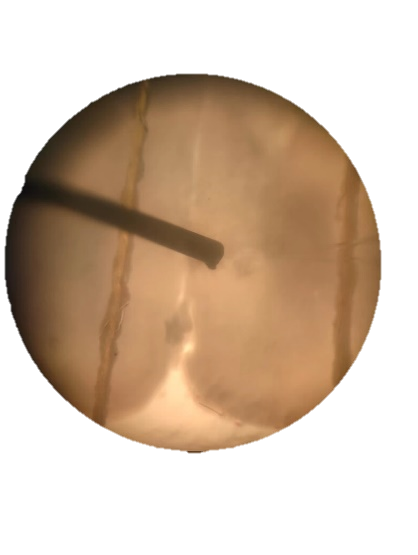

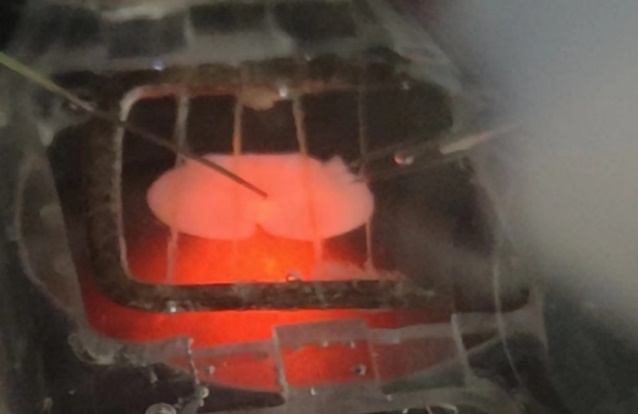


**Figure S16. Physical image of optic fiber position.**

**Table S1.** **Geometry and vibrational frequencies of the ground-state S0 of glutamate with solvent effect obtained by PBE0/6-311G***


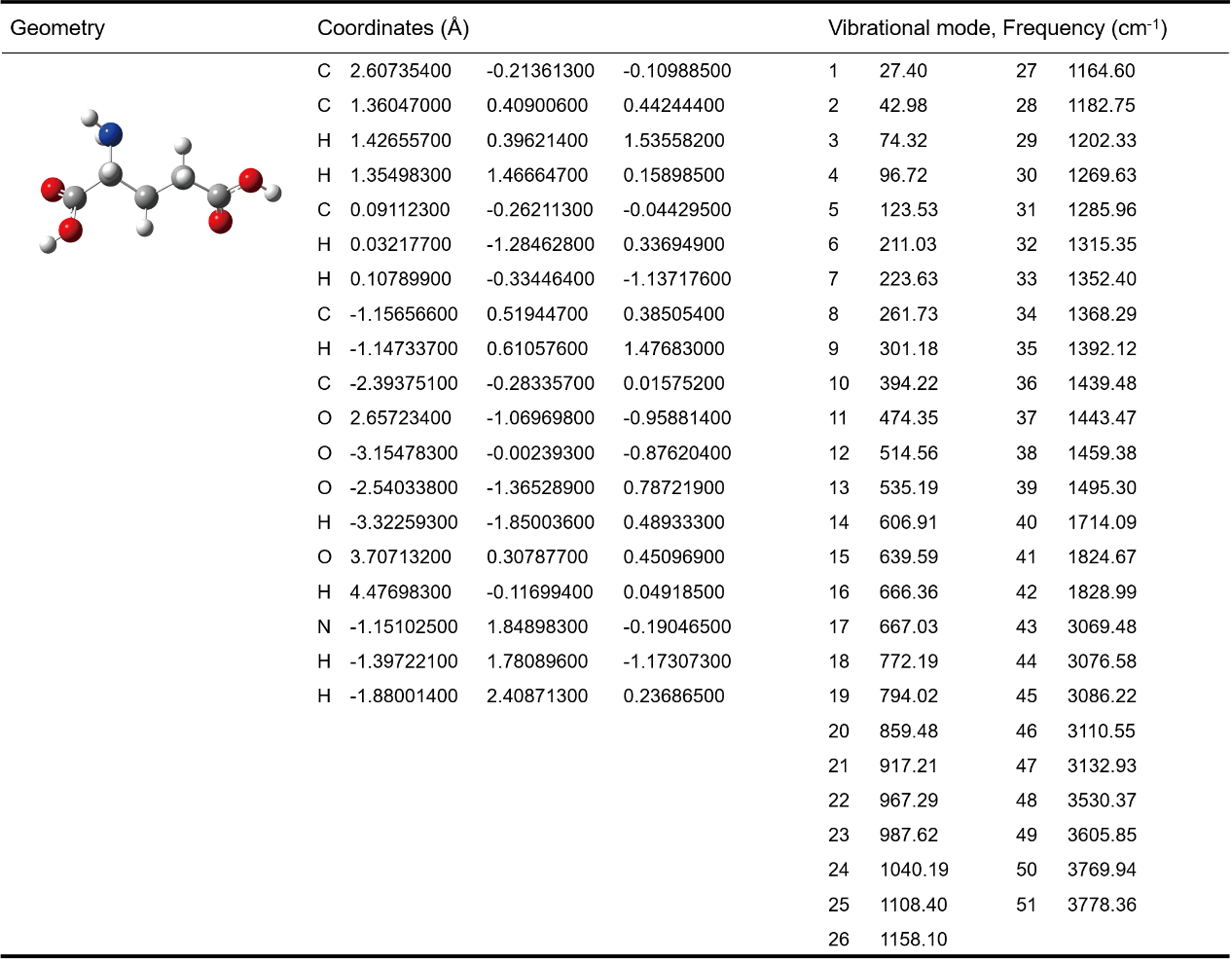


**Table S2.** **Geometry and vibrational frequencies of the excited state S1 of glutamate with solvent effect obtained by PBE0/6-311G***


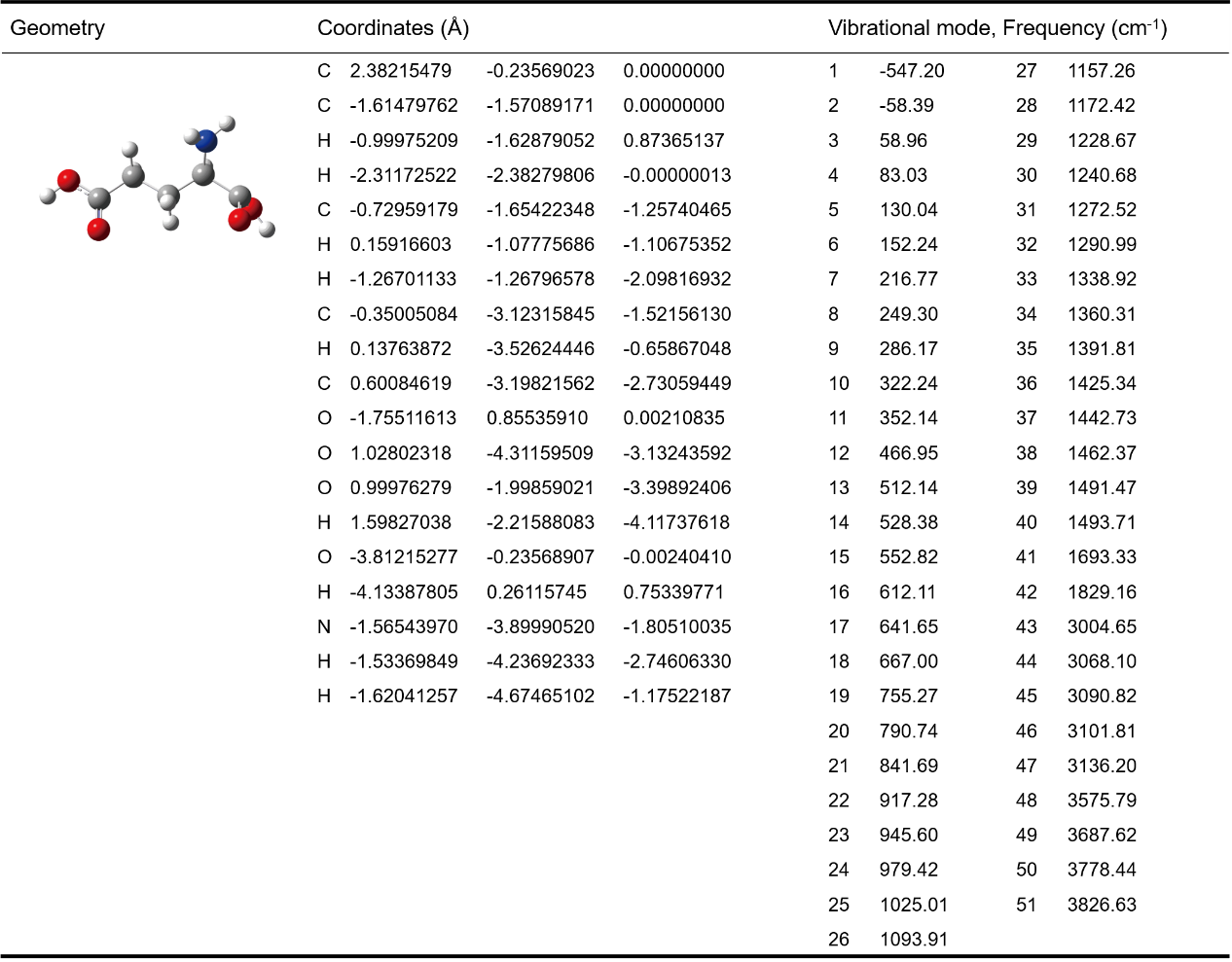


**Table S3.** **Geometry and vibrational frequencies of the ground-state S0 of GABA with solvent effect obtained by PBE0/6-311G***


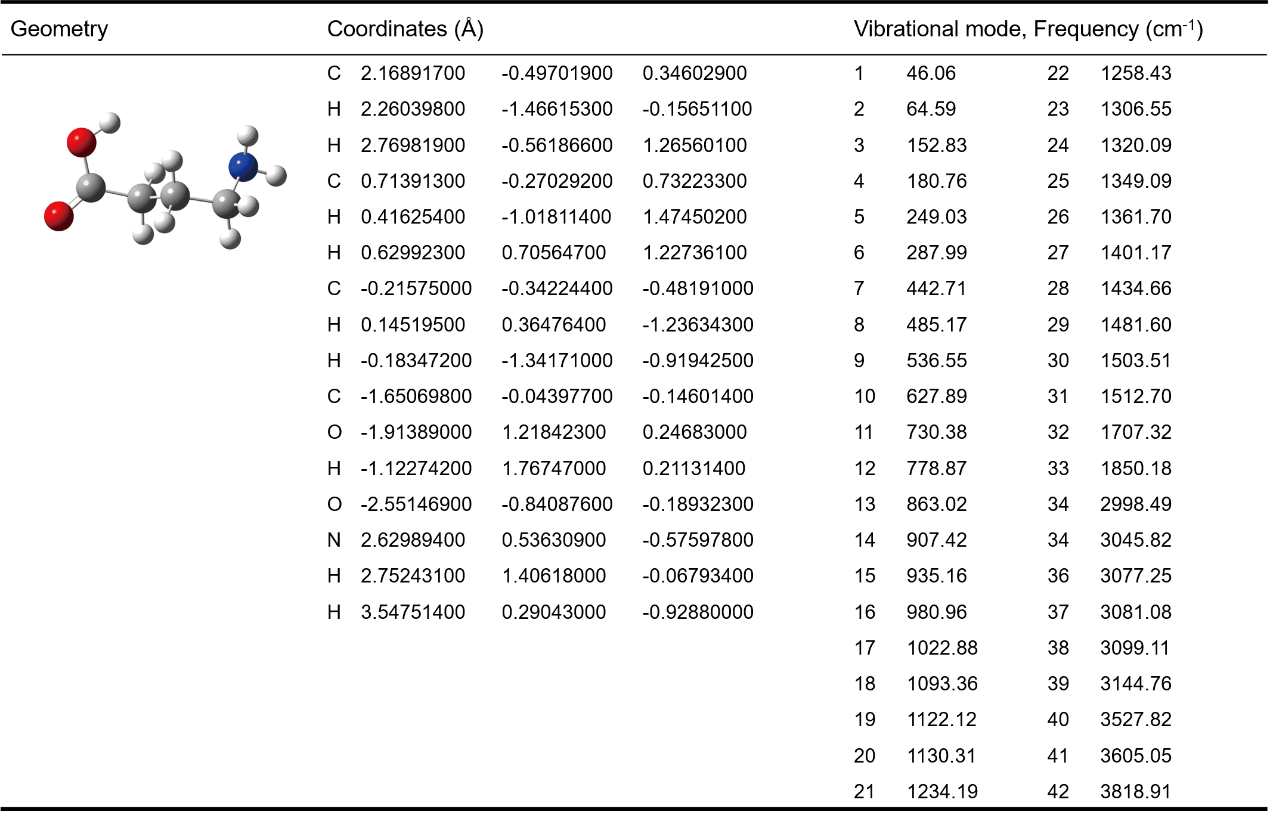


**Table S4.** **Geometry and vibrational frequencies of the excited state of GABA with solvent effect obtained by PBE0/6-311G***


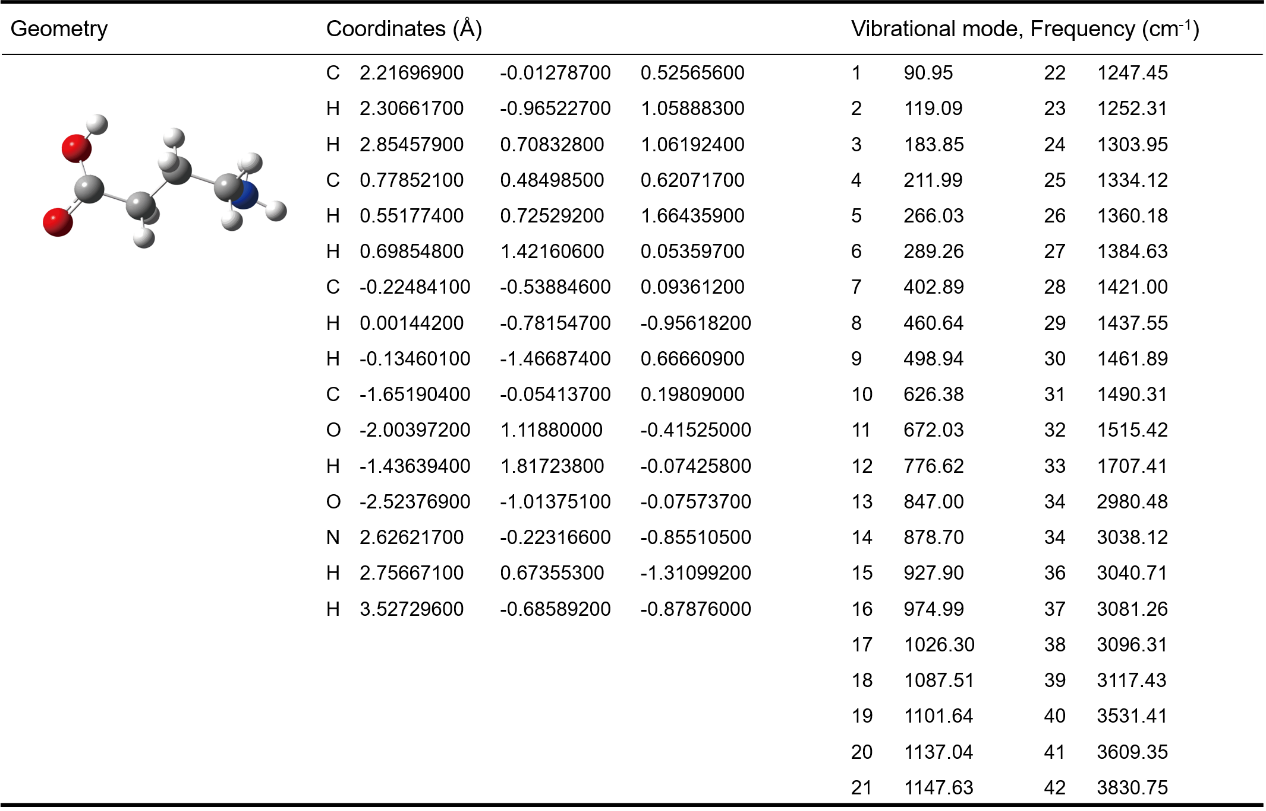


**Table S5. Formula of artificial cerebrospinal fluid and sucrose slicing fluid.**


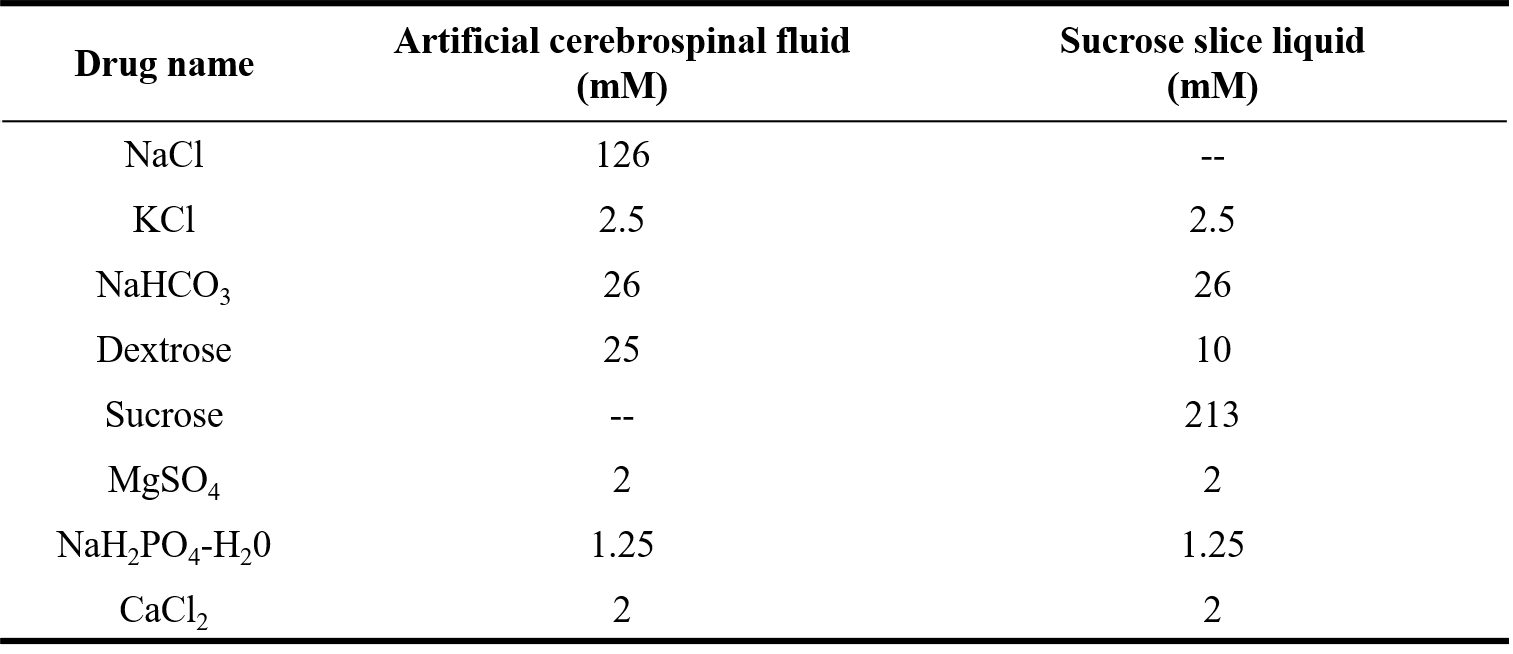


Note: The extracellular fluid is 10×, stored in a 1L volumetric flask at 4℃ refrigerator, on the day of use, dilute to 1× with double steam water, add 2 mL of pre-prepared 1M MgSO_4_ and 1M CaCl_2_ each per liter, and the final concentration is 2 mM. 2L of sucrose slicing solution was prepared at one time, and 4 mL of pre-prepared 1M MgSO_4_ and 1M CaCl_2_ were added, with a final concentration of 2 mM. After the mixture was rushed to adjust pH value until the precipitation is eliminated, then store it in a 2L volumetric bottle in a refrigerator at 4℃ for later use.

**Table S6. Electrode fluid formula.**


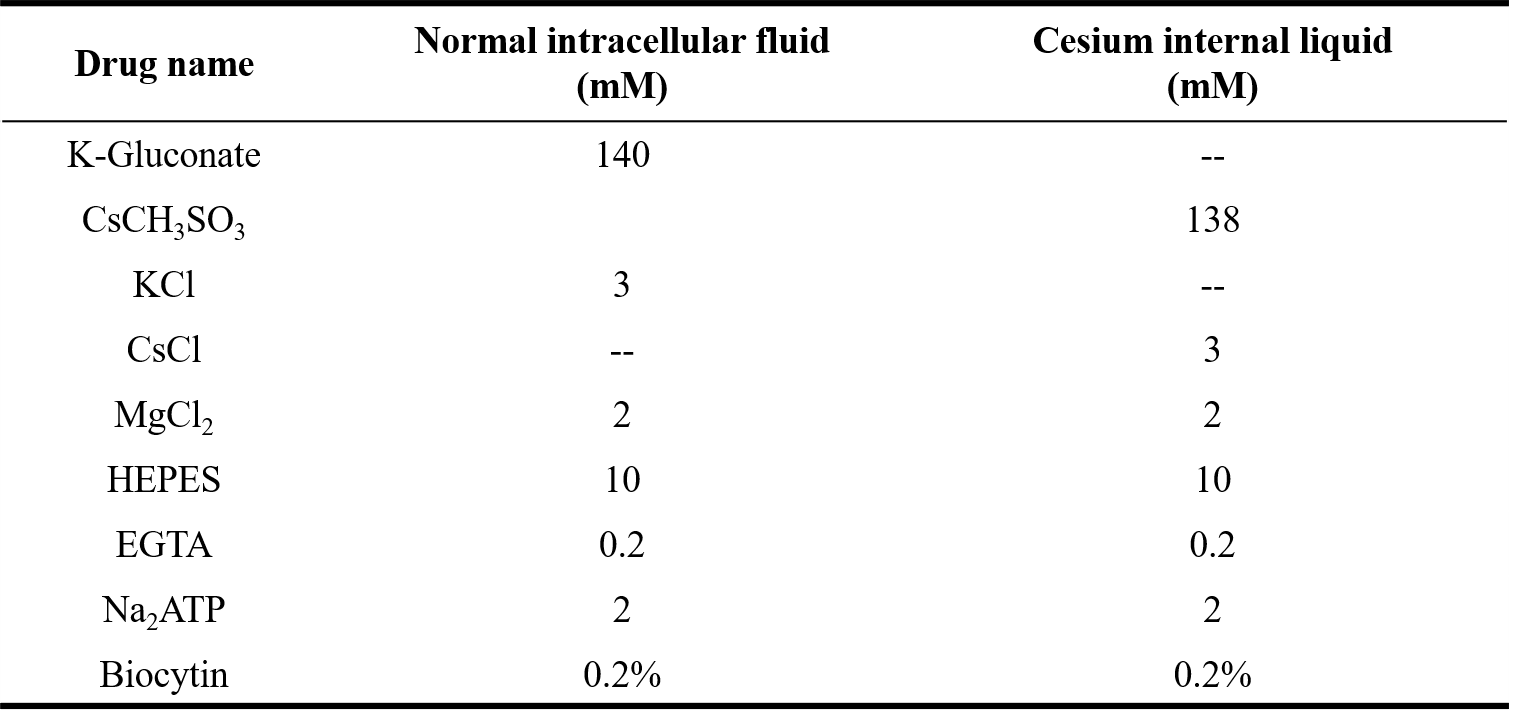


Electrolyte pH: 7.20; osmotic pressure: 280-290 mOsm

References

[1] C. Zhang, Y. Yuan, K. Wu, Y. Wang, S. Zhu, J. Shi, L. Wang, Q. Li, X. Zuo, C. Fan, C. Chang, J. Li, Driving DNA Origami Assembly with a Terahertz Wave. *Nano Lett.* 22, 468-475 (2022).

[2] X.Tan, K.Wu, S.Liu, Y.Yuan, C. Chang, W. Xiong. Minimal-invasive enhancement of auditory perception by terahertz wave modulation. *Nano Res*. S12274-022-4127-7 (2022).

[3] Krishnan R, Binkley J S, Seeger R, Pople J. A. Self-consistent molecular orbital methods. XX. A basis set for correlated wave functions. *J. Chem. Phys.* 72, 650-654 , 10.1063/1.438955 (1980).

[4] Adamo C , Cossi M , Barone V . An accurate density functional method for the study of magnetic properties: the PBE0 model[J]. *J. Mol. Struct*, 493(1-3):145-157 (1999).

[5] Gaussian 16, Revision B.01, Frisch, M. J. *et al.* *Gaussian*, Inc., Wallingford CT, 2016.
